# Editorial

**DOI:** 10.3897/zookeys.930.53478

**Published:** 2020-04-28

**Authors:** Zoltán Korsós, László Dányi

**Affiliations:** 1 Hungarian Natural History Museum, Budapest, Hungary Hungarian Natural History Museum Budapest Hungary

## Abstract

Editorial

It was a great honour when the International Society for Myriapodology (Centre International de Myriapodologie) accepted our proposal to organize the 18th International Congress of Myriapodology at the Hungarian Natural History Museum in Budapest, between August 25–31, 2019. We had a successful meeting: 92 scientific participants (plus 13 accompanying persons) from 32 countries, all around the world from Brazil and Uruguay through Europe and South Africa to China and Australia. During the four scientific days we had 48 lectures (including four invited plenary presentations – one each morning) and 55 posters, which covered a wide range of our field, myriapodology, from the zoological subjects of morphology, anatomy, taxonomy, systematics, physiology, biogeography and nature conservation. We also had a joint full day excursion to the nearby Buda Hills, where participants could encounter some representatives of the local soil fauna. In the name of the organizing committee we would like to thank our 16 Hungarian colleagues who helped to make the congress really enjoyable.

From the presentations, we are presenting here 11 scientific papers in this Special Issue of Zookeys. We are grateful to Pensoft Publishers and to the editorial board of Zookeys for providing the opportunity to publish a cross-section of the 18th International Congress of Myriapodology.

Dr. Zoltán Korsós and Dr. László Dányi

Guest editors

